# Immunotherapy in Ovarian Cancer

**DOI:** 10.1007/s00005-022-00655-8

**Published:** 2022-08-09

**Authors:** Natalia Siminiak, Rafał Czepczyński, Mikołaj Piotr Zaborowski, Dariusz Iżycki

**Affiliations:** 1 Department of Endocrinology and Metabolism, Poznań University of Medical Sciences, Poznań, Poland; 2 Department of Cancer Immunology, Poznan University of Medical Sciences, Poznań, Poland; 3 Department of Gynecology, Obstetrics and Gynecologic Oncology, Division of Gynecologic Oncology, Poznan University of Medical Sciences, Poznań, Poland

**Keywords:** Ovarian cancer, Immunotherapy, Adoptive transfer, Vaccine

## Abstract

Despite advances in surgery and chemotherapy, ovarian cancer remains one of the most lethal malignancies. Hence, the implementation of novel treatment approaches is required to improve the outcomes of the disease. Immunotherapy has been proven to be effective in many tumors and has already been incorporated into clinical practice. In this review, we describe key strategies in immunotherapy of ovarian cancer and summarize data from clinical studies assessing immunological prospects which could improve ovarian cancer treatment approaches in the future. The most notable current strategies include checkpoint blockade agents, the use of vaccines, adoptive cell transfer, as well as various combinations of these methods. While several of these options are promising, large controlled randomized studies are still needed to implement new immunotherapeutic options into clinical practice.

## Introduction

Ovarian cancer is the most lethal malignancy of all gynecological cancers (Bowtell et al. 2015; Cannistra 2004; Sung et al. 2021). Patients are often diagnosed in their advanced stages, due to the lack of initial symptom specificity and screening methods. When it comes to their origin, the vast majority of ovarian neoplasms develop from epithelial tissues (Ledermann et al. 2014) and there are various histology subtypes. High-grade serous ovarian carcinoma (HGSOC) is the most common epithelial subtype (around 75%) and it is highly aggressive with a predisposition to early chemotherapy resistance. HGSOC presents with various molecular abnormalities, especially TP53 mutations observed in > 95% of tumors (Ahmed et al. 2010). In contrast, low-grade serous ovarian carcinoma has a low proliferative rate and the most common mutations are in PTEN/PI3K, RAS, and WNT genes. Identification of histological subtypes and genome features decides about the choice of maintenance therapy following first-line chemotherapy (Lheureux et al. 2019).

The most common treatment approach is based on cytoreductive surgery, commonly combined with chemotherapy (Armstrong et al. 2021). The indication of adjuvant therapy is based on the stage and grade of the tumor, however, only a small group of patients with well-differentiated tumors confined to ovaries do not require systemic treatment. First-line chemotherapy is often a combination of platinum (carboplatin or cisplatin) and taxane (paclitaxel) compounds (Katsumata et al 2013). The neoadjuvant therapy is applied when the optimal cytoreduction surgery is not possible (Wright et al. 2016).

Following systemic chemotherapy, there are options of maintenance treatment. Anti-angiogenic agent, bevacizumab, which affects vascular endothelial growth factor (VEGF) is clinically used in combination with chemotherapy in adjuvant and recurrence treatment (Poveda et al. 2015). In turn, inhibitors of the poly ADP ribose polymerase (PARP) enzyme, such as olaparib or niraparib, are effective in patients with homologous recombination deficit, especially in a group with BRCA mutations (González-Martín et al. 2019; Ledermann et al. 2014; Moore et al. 2018). What is more, studies confirmed significant progression-free survival benefit of bevacizumab and olaparib combination in the group of patients with homologous recombination deficit (Ray-Coquard et al. 2019).

Unfortunately, the risk of disease recurrence remains high after the first-line treatment. Hence, it is necessary to find effective and safe treatment methods, which will deliver not only a complete response but also less toxicity, resulting in diminished side effects. Immunotherapy is a widely researched and innovative strategy, which could soon dominate systematic chemotherapy (Levinson et al. 2019; Lynam et al. 2020). Active immunotherapy uses the immune system to recognize and target specific cancer antigens, for example, vaccines, which stimulate the patient’s immune response, or chimeric antigen receptor T-cell (CAR-T) therapy; collected from the patient and genetically engineered immune cells with the ability to recognize antigens, or target therapy; a specific antibody designed to eliminate the specific target. Passive immunotherapy enhances the activity of a patient’s immune system response, for example, checkpoint inhibitors or cytokines. Immunomodulatory therapy that blocks the suppressive signals of checkpoint proteins (checkpoint blockade), or selectively targets immunosuppressive cells in the tumor microenvironment (such as Tregs), allows for activation and proliferation of tumor-specific T cells, which are able to identify and eliminate cancer cells.

However, in clinical trials, immunotherapy still does not provide a sufficient response rate. Moreover, dysregulation of the immune system caused by this approach might result in immune-related adverse effects. Hence, when introducing immunotherapy, it is necessary to establish a suitable treatment regimen and a possible combined therapy, as well as manage the potential side effects (Levinson et al. 2019; Lynam et al. 2020; Palaia et al. 2020).

## Checkpoint Blockade

Immunological checkpoint inhibitors are the most promising prospective therapy for incurable tumors, including ovarian cancer.

When an effector T-cell binds a specific ligand on a tumor cell, immune checkpoints (co-signaling pathways that modify T-cell receptor (TCR) signaling) may enhance or suppress the immune response. Immune checkpoints act as a negative feedback mechanism, modulating effector cell response to protect the host against autoimmunity and maintain self-tolerance. These pathways play an important role during tumorigenesis, as they are the main mechanism of tumor cell immune resistance. The best-known and widely used checkpoints include T-cell surface molecules, such as cytotoxic T-lymphocyte antigen 4 (CTLA-4) and programmed death 1 (PD-1) (Friese et al. 2020; Hamanishi et al. 2015; Robert et al. 2014; Wieser et al. 2018).

The tumor-specific immune response is activated after the recognition of cancer cell-specific antigens. Antigen-presenting cells (APCs), similarly to dendritic cells, migrate to the lymph nodes and present antigens to T cells, which in turn are responsible for attacking cancer via TCRs (Chen and Flies 2013).

Immune checkpoints modify T-cell response, suppressing or stimulating immunity during antigen presentation. For example, the interaction between CD28 on T cells and B7 protein on APC cells is responsible for the initiation of the immune response. In turn, CTLA-4 negatively regulates the early phase of T-cell activation (Chen and Flies 2013; Hamanishi et al. 2016).

These immune mechanisms are also present in the local environment of cancer. The interaction of PD-1 protein on T cells and programmed cell death-ligand 1 (PD-L1) protein on cancer cells causes attenuation of T-cell action. In patients with ovarian cancer, PD-L1 expression is associated with a worse prognosis and is correlated with the suppression of local immune response (Zamarin et al. 2020).

The mechanism of immunotherapy is based on blocking natural immune response inhibitor pathways, enabling T cells to remain active and eliminate cancer cells.

The response to immunotherapy, such as anti-PD-1/PD-L1, depends on the heterogeneity of the tumor microenvironment, which may be characterized as cold (noninflamed) or hot (inflamed). Hot tumors show higher T-cell infiltration and activity level than cold tumors, resulting in a better response to immunotherapy. Researchers are investigating whether combined therapies and stimulation of the tumor’s immune system can enhance the response to immunotherapy (Duan et al. 2020).

### CTLA-4 Inhibitors—IPILIMUMAB

CTLA-4 inhibitors are immune checkpoint blockers stimulating the immune response. Ipilimumab, the anti-CTLA-4 antibody, is mostly used in treatment of malignant melanoma (Hodi et al. 2010). However, dose-dependent side effects often develop within the first few weeks to months after treatment, affecting the skin, gastrointestinal tract, liver, and endocrine system. The immune toxicity appears more frequently and more severely during administration of ipilimumab compared to PDL-1 inhibitors (Fecher et al. 2013).

### Antibodies Against PD-1/PD-L1

Antibodies against PD-1/PD-L1 are successfully used in treatment of malignant melanoma, non-small-cell lung cancer, and urothelial cancer.

The blockade of the PD-1 inhibitor pathway is being clinically explored and has shown less immunotoxicity than ipilimumab. The side effects are similar, but occur less frequently and are less severe. The only exception is a higher risk of pneumonitis. However, monotherapy with nivolumab presented a low tumor response in ovarian cancer (Hamanishi et al. 2015). Pembrolizumab administered as a single agent was effective only in a small fraction of patients with recurrent ovarian cancer (Matulonis et al. 2019). The overall response rate ranged 7–9%, depending on the number of previous platinum regimens (Matulonis et al. 2019).

### Combination Therapies

Combining immunotherapies that exploit different mechanisms may have a complementary effect. Combination therapy with anti-CTLA-4 and anti-PD-1 delivered promising results, demonstrating greater efficacy compared to monotherapy (Zamarin et al. 2020). Higher rate of response and greater prolongation of progression-free survival was described, with more patients remaining progression-free at six months after initiation of therapy. Unfortunately, while a higher incidence of side effects has been described compared to monotherapy with nivolumab, most immune-related adverse effects were manageable. However, it is always important to carry out detailed monitoring of the patient and react to any potential signs and symptoms of toxicity. Moreover, a pretreatment assessment of the patient should be performed for early recognition of potential predisposition to side effects (Zamarin et al. 2020).

Therapies with a primary non-immune mechanism of action may make tumors susceptible to immunotherapy. PARP inhibitors that lead to DNA damage in BRCA1-deficient ovarian cancer cells, trigger an antitumor immune response (Ding et al. 2018). This effect can be enhanced by anti-PD1 treatment, which provides a rationale for combining PARP inhibitors and immunotherapy (Ding et al. 2018). In the clinical study, PARP inhibitor—niraparib has been shown effective in combination with the anti-PD-1 antibody, pembrolizumab in recurrent platinum-resistant ovarian cancer (Konstantinopoulos et al. 2019). VEGF plays an important role in angiogenesis and dissemination in ovarian cancer. Interestingly, VEGF also inhibits dendritic cells and tumor-infiltrating lymphocytes (Chen and Hurwitz 2018). The combination of VEGF inhibitor—bevacizumab with pembrolizumab has been shown safe and effective in ovarian cancer in phase 2 clinical study (Haunschild and Tewari et al. 2020; Zsiros et al. 2021). An ongoing clinical trial evaluates also pembrolizumab in combination with pegylated liposomal doxorubicin in platinum platinum-resistant ovarian cancer (Lee et al. 2020; Park et al. 2022).

However, one of the main challenges is to identify a biomarker, which may predict the efficiency of immunotherapy and indicate which patient will most likely respond to treatment. Currently, the expression of PD-L1 is used as the most predictive marker (Wang 2019). It is measured as the percentage of tumor cells that express PD-L1, or a number of PD-L1 positive tumor cells, lymphocytes, and macrophages divided by the total number of viable tumor cells. PD-L1 positivity evaluation is recommended in non-small cell lung cancer, metastatic urothelial cancer, gastric cancer, and cervical cancer. Higher PD-L1 expression (combined positive score > 10) was also predictive of a better response to pembrolizumab therapy in ovarian cancer (Matulonis et al. 2019). Nevertheless, the cut-off value, which may be used in ovarian cancer, is still under investigation (Pawłowska et al. 2021; Wang 2019). In advanced or recurrent endometrial cancer, the susceptibility to anti-PD1 therapy is determined based on hallmarks of microsatellite instability (Oaknin et al. 2020). It is a condition that leads to the increase of DNA replication errors that result in the generation of new aberrant cell surface proteins. Those neoantigens are well recognized and rejected by the host immune response. At some point, however, cancer cells suppress this effect via PD-L1 signaling. For this reason, anti-PD1 molecules, such as dostarlimab or pembrolizumab, are effective in restoring antitumor response (Marabelle et al. 2020; Oaknin et al. 2020). The status of microsatellite instability is determined based on the immunohistochemical staining for proteins involved in mismatch repair (MLH1, PMS2, MSH2, MSH6). This assay serves as a biomarker to identify responders to the anti-PD1 immunotherapy. In contrast to endometrial cancer, however, microsatellite instability is rare in ovarian cancer (< 2%) (Bonneville et al. 2017). Another approach is to assess the status of antitumor response based on biomarkers in peripheral blood. For instance, the activity of cytotoxic cells was determined by measuring the expression of granzyme B in peripheral blood mononuclear cells (Zaborowski et al. 2021). This assay revealed that many patients with ovarian cancer have suppressed cytotoxic responses. The effect was even more pronounced in higher-stage diseases. There is a need for new biomarkers to predict response to immunotherapy and to monitor the treatment of ovarian cancer.

## Adoptive Cell Transfer

Lymphocytes, either derived from autologous tumor tissue or engineered to target tumor-specific antigens, can be infused to help the immune system of cancer patients. This usually requires cell activation and expansion ex vivo. Leukapheresis is applied to isolate tumor-reactive effector cells, which are subsequently primed in culture using immunomodulatory agents to promote their survival and differentiation (Levinson et al. 2019). The first clinical trial testing this approach has been completed in patients with metastatic melanoma (Rosenberg et al. 1988).

In patients with ovarian cancer, early phase I and II clinical trials have been performed, assessing the use of tumor-infiltrating lymphocyte (TIL) adoptive transfer for advanced-stage disease. The results demonstrated a substantial duration of response compared to conventional chemotherapy (Aoki et al. 1991). Fujita et al. (1995) completed a study on 13 women treated with T-lymphocyte infusion after undergoing surgery followed by chemotherapy and demonstrated an increased three-year disease-free survival rate. Furthermore, intraperitoneal T-lymphocyte infusion has also been evaluated but with less promising results (Kershaw et al. 2006). An important limitation of these early trials includes the lack of pretreatment lymphodepletion therapy, which may have negatively impacted results.

Recently, clinical studies focus mostly on the evaluation of adoptive cell transfer (ACT) in combination with other therapeutic options, including checkpoint blockade (Kverneland et al. 2020; Sarivalasis et al. 2021). For instance, Kverneland et al. (2020) reported promising results of one patient with partial response and five patients achieving prolonged disease stabilization, after receiving ACT combined with ipilimumab and nivolumab. Before surgery patients received ipilimumab to activate T cells immune response—the increased expansion of TILs in the tumor was confirmed ex vivo, and the infusion of nivolumab was performed after tumor resection.

### CAR-T Cell Therapies

CAR-T cells are genetically engineered, patient-derived, white blood cells, which are programmed to identify tumor-cell-surface antigens and activate specific immune response.

CAR-T-cell therapy has developed in recent years and has proven effective in hematological malignancies, but similar results have not been reported in solid tumors (Ruella and Kenderian 2017). The most difficult problem is identifying specific antigens that are overexpressed in tumors and not in non-pathological tissues. The most common target antigens in ovarian cancer CAR-T include MUC16, mesothelin, HER2 and folate receptor α (FRα) (Yan et al. 2019). Chekmasova et al. (2010) confirmed that MUC16-CAR-T cells may delay progression in mouse models. Mesothelin is overexpressed in a variety of cancers, including ovarian cancer, but it is also expressed in non-pathological tissues, which can cause off-target effects. Clinical trials with mesothelin-targeting CAR-T cells are ongoing in patients with mesothelioma, lung cancer and breast cancer, also evaluating combination therapy with pembrolizumab. Neelapu et al. (2018) presented results of four patients with pancreatic cancer, who did not occur serious reactions to mesothelin-CAR-T cells. Despite great potential of CAR-T-cell therapy and its success in hematology, there are no satisfactory effects in solid tumors and ovarian cancer. The various tumors microenvironments and antigens expression enable to achieve sufficient response. Possibly, combined therapies will improve CART-T anticancer activity by stimulating tumor immune cells infiltration.

## Therapeutic Cancer Vaccines

Unlike traditional cytotoxic therapies, vaccine-induced immune responses inhibit disease tumor growth and/or recurrence using modulated immune responses. For example, peptide vaccines and dendritic cell therapies can activate the patient’s anticancer immunity system. Furthermore, several vaccines, including mutated p53 peptides, NY-ESO-1, and mesothelin were already investigated in ovarian cancer (Ledermann et al. 2013).

While genetic abnormalities of the p53 protein have been observed in most advanced ovarian cancer patients, the p53 vaccine did not provide enough improvement in subsequent chemosensitivity or progression-free survival (Leffers et al. 2009; Rahma et al. 2012).

The cancer-testis/cancer-germline antigen, named New York esophageal squamous cell carcinoma-1 (NY-ESO-1) has been shown to be present in numerous cancer cell types, including epithelial ovarian cancer, which indicates it as a potential vaccine target (Odunsi et al. 2003). In addition, the expression of NY-ESO-1 in epithelial ovarian cancer was associated with phenotypically aggressive disease, and it has been shown that the expression of this antigen significantly reduces overall survival (Szender et al. 2017). Furthermore, several studies have been performed to evaluate the possible effect of NY-ESO-1 vaccination in ovarian cancer patients. The results demonstrated vaccine-induced CD4^+^ and CD8^+^ T-cell responses, as well as the persistence of NY-ESO-1^+^ lymphocytes (Davis et al. 2004; Odunsi et al. 2003, 2007). Moreover, a small clinical study suggested a survival benefit among NY-ESO-1 vaccinated compared to non-vaccinated patients (Odunsi et al. 2012). Administration of demethylation agents in conjunction with NY-ESO-1 vaccination resulted in some degree of clinical response (partial response or stable disease) in 6 of 10 patients, as well as significant NY-ESO-1^+^ lymphocyte response (Odunsi et al. 2012). Similarly, it has been shown that the NY-ESO-1 synthetic overlapping long peptide vaccine is safe and rapidly induces consistent integrated immune responses in nearly all vaccinated patients (Sabbatini et al. 2012). Another phase I study by Diefenbach et al. (2010), assessing vaccination of patients with epithelial ovarian cancer in high-risk first remission with the HLA-A*0201-restricted NY-ESO-1b peptide, showed induction of specific T-cell immunity. In addition, three of nine patients remain in complete clinical remission at 25, 38, and 52 months after treatment (Diefenbach et al. 2010).

Implementation of dendritic cell-based vaccines is yet another approach to treatment, also being investigated in patients with ovarian cancer. Vaccination with the autologous dendritic cell-based vaccine with whole tumor lysate after systemic chemotherapy resulted in a decrease in progression rate, as well as improved overall survival in ovarian cancer (Tanyi et al. 2018). In turn, in a phase II study by Gray et al. (2016), evaluating mucin 1 targeted-dendritic cell treatment for maintenance therapy in recurrent ovarian cancer, improved overall survival has been observed in vaccinated patients compared to controls. Vaccination with dendritic cells pulsed with autologous tumor cell lysate supernatants has been suggested to be beneficial and warrant a large-scale clinical trial (Kandalaft et al. 2013). A phase I study involving dendritic cells pulsed with FRα showed induction of IL-17 producing T cells and demonstrated the recurrence-free time of 49 months in 7 out of 18 patients (Block et al. 2020).

The assessment of the clinical use of vaccines in cancer patients has certain limitations (Friese et al. 2020; Levinson et al. 2019). Firstly, surgical resection of adequate tumor samples to synthesize cell-based vaccines is needed. Secondly, the heterogeneity of antigen expression within a tumor, as well as recognition of limited epitopes for a given tumor antigen, are both potentially important issues. In addition, in the case of dendritic cells-based vaccines, intensive cell expansion is needed, leading to possible inter-laboratory differences in cell preparation. All of the above might be a reason for less prominent clinical benefits in later phase II and III trials assessing vaccines in patients with ovarian cancer (Liao and Disis 2013).

## Side Effects of Immunotherapy

Therapeutic enhancement of immune response may lead to autoimmune disorders. Previous studies have already reported among the most common hypothyroidism, hyperthyroidism, skin rush, and colitis (Matulonis et al. 2019). An emergence of ovarian cancer can induce a paraneoplastic autoimmune reaction that may precede clinical diagnosis of malignancy. Those disorders may include, for instance, dermatomyositis and paraneoplastic neurological syndromes (Requena et al. 2014; Zaborowski et al. 2015). It has already been observed that immunotherapy may also induce those conditions (Valencia-Sanchez and Zekeridou, 2021). For example, severe encephalitis affected a patient with recurrent clear cell ovarian cancer treated with nivolumab (anti-PD1 immunotherapy) (Burke et al. 2018).

## Conclusion

**Fig. 1:**
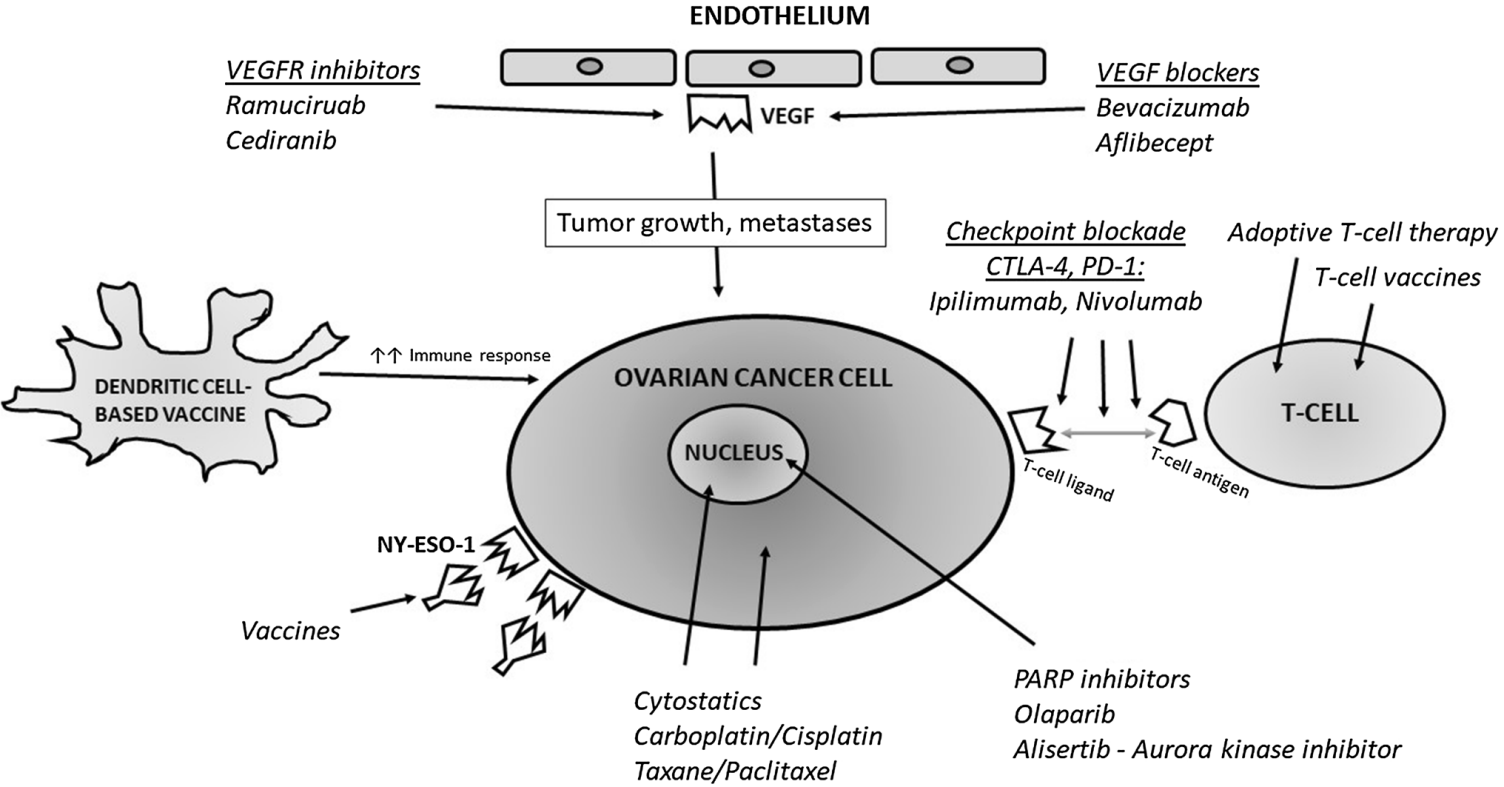
Current immunotherapy approaches to ovarian cancer treatment. Description in the text

Even though the immune system has a crucial role in the pathogenesis of ovarian cancer, the clinical application of immunotherapy has been limited to small pilot studies in ovarian cancer. The main directions for the development of therapeutic approaches in patients with ovarian cancer include the checkpoint blockade, vaccination-based approaches, as well as adoptive cell transfer (Fig. 1). However, despite promising results of small pilot studies, clinical use of immunotherapy in ovarian cancer has still not been implemented, mostly due to insufficient experimental evidence of their effectiveness. Better understanding of key biological mechanisms, along with future technological developments, will most likely be the key to expanding the use of immune therapeutics and subsequently improving patients’ clinical outcomes.

## Data Availability

This manuscript has no associated data or the data will not be deposited. This is a review.
